# PROLONG: penalized regression for outcome guided longitudinal omics analysis with network and group constraints

**DOI:** 10.1093/bioinformatics/btaf099

**Published:** 2025-03-07

**Authors:** Steven Broll, Sumanta Basu, Myung Hee Lee, Martin T Wells

**Affiliations:** Department of Statistics and Data Science, Cornell University, Ithaca, NY 14850, United States; Department of Statistics and Data Science, Cornell University, Ithaca, NY 14850, United States; Department of Medicine, Weill Cornell Medicine Graduate School of Medical Sciences, New York, NY 10065, United States; Department of Statistics and Data Science, Cornell University, Ithaca, NY 14850, United States

## Abstract

**Motivation:**

There is a growing interest in longitudinal omics data paired with some longitudinal clinical outcome. Given a large set of continuous omics variables and some continuous clinical outcome, each measured for a few subjects at only a few time points, we seek to identify those variables that co-vary over time with the outcome. To motivate this problem we study a dataset with hundreds of urinary metabolites along with Tuberculosis mycobacterial load as our clinical outcome, with the objective of identifying potential biomarkers for disease progression. For such data clinicians usually apply simple linear mixed effects models which often lack power given the low number of replicates and time points. We propose a penalized regression approach on the first differences of the data that extends the lasso + Laplacian method [Li and Li (Network-constrained regularization and variable selection for analysis of genomic data. Bioinformatics 2008;24:1175–82.)] to a longitudinal group lasso + Laplacian approach. Our method, PROLONG, leverages the first differences of the data to increase power by pairing the consecutive time points. The Laplacian penalty incorporates the dependence structure of the variables, and the group lasso penalty induces sparsity while grouping together all contemporaneous and lag terms for each omic variable in the model.

**Results:**

With an automated selection of model hyper-parameters, PROLONG correctly selects target metabolites with high specificity and sensitivity across a wide range of scenarios. PROLONG selects a set of metabolites from the real data that includes interesting targets identified during EDA.

**Availability and implementation:**

An R package implementing described methods called “prolong” is available at https://github.com/stevebroll/prolong. Code snapshot available at 10.5281/zenodo.14804245.

## 1 Introduction

An emerging problem in omics research in the clinical domain is the discovery of useful biomarkers for repeated-measure data where both the clinical outcome and a large number of omics predictors are measured at a few times on a small number of individuals. In many clinical studies of disease progression, prognosis, and effectiveness of new treatments, we track clinical outcomes of a small group of patients over a handful of times (a few weeks, t=3−8). Recently it is also more feasible to collect data on a large number of omics features such as metabolite abundance or gene expressions. Clinicians with access to this large pool of omics features are interested in identifying biomarkers associated with the clinical outcome.

**Figure 1.**
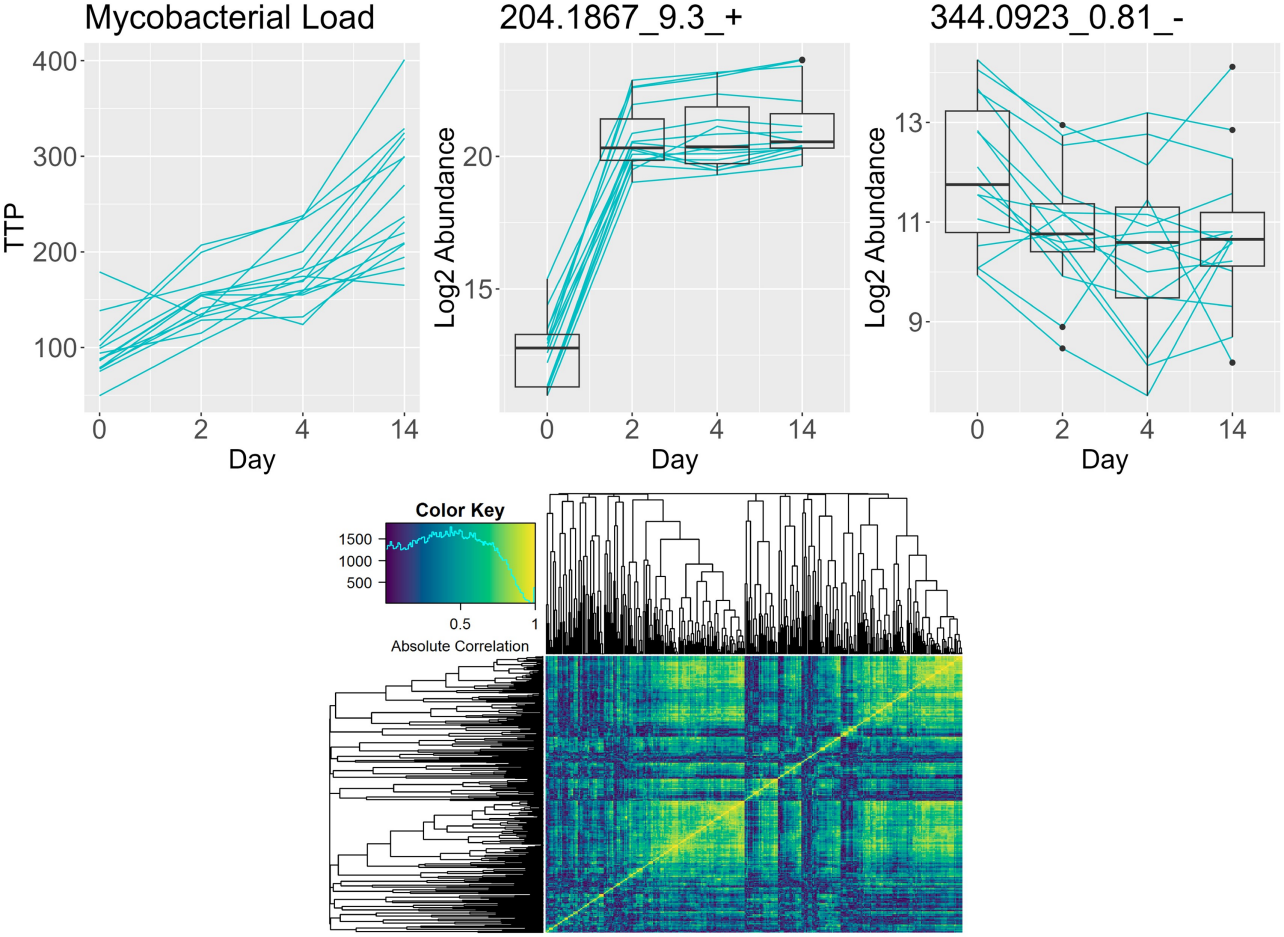
Trajectories for time to positivity which is inversely related to mycobacterial load (top left), an interesting metabolite (top center), and a more stationary but still potentially interesting metabolite (top right). Absolute correlation heatmap for all metabolites for difference of the first two time points (bottom).

Our work is motivated by a pulmonary tuberculosis study consisting of 4 time point observations for 15 subjects each with measured sputum mycobacterial load, quantified by the time to positivity (TTP) in a *Mycobacterium tuberculosis* (Mtb) culture, and abundances of 352 urinary metabolites. These metabolites are of interest for their potential use as non-invasive markers of the disease process and for insight into biological pathways (Cliff *et al.* 2013, Dutta *et al.* 2020, Li *et al.* 2021). Previous work has been performed on the identification of urinary metabolite-based biomarkers for patients with pulmonary tuberculosis (Isa *et al.* 2018, Xia *et al.* 2020) and the creation of a general methodology for metabolite-based biomarker discovery in settings with longitudinal continuous outcomes. Figure 1 shows trajectories over time for the Mtb TTP and for two metabolites. The TTP (top left) shows a consistent upward mean shift, but the individual subject trajectories often show dips. Despite a small number of time points and subjects, we can clearly see associations between the outcome and the metabolite trajectories.

Existing works in the short panel clinical omics literature often use univariate longitudinal linear mixed effects (LMEs) regression models (Walsh *et al.* 2020, Wipperman *et al.* 2021) for the clinical outcome of interest. Typically, variable selection is conducted by comparing the *P*-values of the univariate models. Beyond the usual multiplicity adjustment limitations of one-at-a-time modeling and testing, we argue that, by failing to incorporate the underlying omic feature and time correlation structures, this approach is susceptible to low sensitivity and statistical power. For our data, LME does not select a single metabolite.

The literature on high-dimensional omics in molecular biology and genomics is vast. However, many of these methods are not designed around using a time-varying clinical outcome, often focusing on differential expression for two or more groups, including DESeq2 (Love *et al.* 2014), limma (Ritchie *et al.* 2015), OmicsLonDA (Metwally *et al.* 2022). Generalized Estimating Equations (GEEs) (Xu *et al.* 2014), have been applied to modeling outcomes with longitudinal omics data, but this approach requires more samples than are present in our data.

We propose the PROLONG method, Penalized Regression for Outcome guided Longitudinal Omics analysis with Network and Group constraints, which can jointly select longitudinal features that co-vary with a time-varying outcome on the first-difference scale, either contemporaneously or with lags. PROLONG combines the group lasso and Laplacian penalties as an extension of the network-constrained regularization model introduced by Li and Li (2008). The group lasso penalty induces sparsity while grouping all of the contemporaneous and lag terms of each variable, and the network penalty incorporates the correlation structure of the data.

The first-differencing of the outcome and omics variables provides multiple benefits over the levels scale for this problem. Most importantly, it increases power over level scale analysis in the same way a paired t-test offers more power than an unpaired t-test when the two groups are positively correlated. We can view its effect in metabolite 344.0923_0.81_- shown in Fig. 1. The metabolite abundance *levels* decrease from day 0 to day 2, but the boxplots have substantial overlap. However, tracking the *changes in levels* for every subject using the blue lines, we see that all except one subject’s metabolite level decreased, increasing our confidence that this change is real. First-differencing also helps us cancel out any time-invariant terms like the intercept and subject-level effects, reducing the number of parameters to estimate and simplifying the model.

The primary innovation of this paper is the use of first-differencing for both the omics predictors and the clinical outcome to gain power in high-dimensional analysis. To our knowledge, first-differencing has not been used in the omics literature despite its ubiquitous use in low-dimensional problems under the so-called change score framework (Oldham 1962, Tu and Gilthorpe 2007, Tennant *et al.* 2022). Additionally, our inclusion of lag effects allows for the selection of omics variables with more complex relationships with the clinical outcome.

We test PROLONG in simulations with both uncorrelated and correlated data, comparing univariate and multivariate versions of PROLONG with LME. LME performs the worst in all scenarios, especially struggling in the first-difference scale simulations. Both versions of PROLONG have near-perfect sensitivity in all scenarios, with the multivariate PROLONG having better specificity as dimension and sparsity increase.

PROLONG is also tested on the motivating TB clinical trials data, shown in Fig. 1. For this data, PROLONG, which itself does not apply FDR control, selects a set of target metabolites identified in exploratory data analysis and by clinicians, while the univariate longitudinal model picks out no metabolites at reasonable FDR thresholds. Some metabolites selected by PROLONG were identified as targets from the exploratory analysis phase due to their sharp change from the baseline to the second measurement, like 204.1867_9.3+. whose trajectories are shown in Fig. 1. However, many trajectories are not cleanly separated between baseline and post-treatment, such as 344.0923_0.81_- shown in Fig. 1. These metabolites have different levels of variation across time points but do not show a sustained long-term effect after treatment, highlighting the flexibility of PROLONG.

## 2 Materials and methods

To provide motivation for PROLONG, we first describe the univariate mixed effects model often used for longitudinal omics regression (Walsh *et al.* 2020). Then we provide our proposed univariate alternative model and a natural hypothesis testing technique. We end this section with our proposed multivariate extension, which includes constraints to induce sparsity in the high-dimension scenario and to incorporate the dependence structure of the omics predictors.

### 2.1 Background

This article’s motivating example is a short-term pulmonary tuberculosis drug trial (Xia *et al.* 2020, Wipperman *et al.* 2021). The prototypical data from such a short-term trial consists of regressors ${\tilde{X}}_{it}^{\left[j\right]}$ and response ${\tilde{Y}}_{it}$ for subject i=1,…,n, omics feature j=1,…,p, and time point t=1,…,T. A univariate LMEs model for the levels of ${\tilde{Y}}_{it}$ then takes the form

(1) $${\tilde{Y}}_{it}=\alpha_{t}^{\left[j\right]}+\beta_{0}^{\left[j\right]}+\beta^{\left[j\right]}{\tilde{X}}_{it}^{\left[j\right]}+b_{i}+\epsilon_{it},$$

where $\alpha_{t}^{\left[j\right]}$ is our mean response across subjects at time *t*, *b_i_* is the subject-specific random effect, *ϵ_it_* is the mean-zero error term, $\beta_{0}^{\left[j\right]}$ is a feature-specific intercept term, and $\beta^{\left[j\right]}$ is the feature-specific parameter of interest.

Longitudinal mixed effects models account for potential temporal dependence in the data as well as subject-level variation. Still, they may have insufficient power to select true metabolites with short-term changes and trends over time in our data setting with such small *n* and *T*. This is particularly relevant when the time course data is primarily focused on short-term dynamics, and controlling for time-invariant unobservable factors is crucial (Wooldridge 2010).

To eliminate time-invariant effects and to pair our observations over time to increase power, we move to the first-difference, or delta, scale for both ${\tilde{Y}}_{it}$ and ${\tilde{X}}_{\mathrm{ijt}}$. When we regress the first-differences of the outcome on our first differences of the metabolites, we seek to identify short-term changes in our outcome that are associated with short-term changes in our feature abundances. This first-difference approach addresses the unobserved time-invariant heterogeneity as well as potential endogeneity concerns related to time-invariant unobserved factors.

The first-difference approach is appropriate for our motivating example, as the subjects all receive the same treatment but show time heterogeneous TTP and metabolite abundances. We view our independent variables not as a treatment themselves but as potential mediators for the treatment effect on the clinical outcome. Identifying significant linear relationships on the delta scale can inform clinicians of biomarkers for early disease progression.

### 2.2 PROLONG: univariate case

In this subsection we give the sequence of step to deduce a matrix representation of the differenced regression model from Equation (1). For each subject, we first matricize the data to incorporate all time points into a single model. Once in the form of matrices, the difference between subsequent time points can be represented in terms of other matrices.

Given our original model in Equation (1), the *n *×* T* response matrix ${\tilde{Y}}_{it}$ can be represented and differenced in the following step

(2) $${\left[\begin{array}{ccc} {\tilde{Y}}_{11} & \cdots & {\tilde{Y}}_{1T}\\ & \vdots & \\ {\tilde{Y}}_{n1} & \cdots & {\tilde{Y}}_{nT} \end{array}\right]}_{n\times T}\;\to \;\;{\left[\begin{array}{ccc} \Delta {\tilde{Y}}_{11} & \cdots & \Delta {\tilde{Y}}_{1(T-1)}\\ & \vdots & \\ \Delta {\tilde{Y}}_{n1} & \cdots & \Delta {\tilde{Y}}_{n(T-1)} \end{array}\right]}_{n\times (T-1)},$$

where each $\Delta {\tilde{Y}}_{it}$ is the first-difference ${\tilde{Y}}_{i(t+1)}-{\tilde{Y}}_{it}$.

Vectorizing this matrix yields the n(T−1)×1 vector *Y* to be used as the dependent variable in our differenced model where

(3) $$Y={\left[\Delta {\tilde{Y}}_{11},\ldots ,\Delta {\tilde{Y}}_{n1}\ldots ,\Delta {\tilde{Y}}_{1(T-1)},\ldots ,\Delta {\tilde{Y}}_{n(T-1)}\right]}^{⊤}.$$

For the jth metabolite (j=1,…,p), the n×T matrix can be represented and differenced in the following step

(4) $${\left[\begin{array}{ccc} {\tilde{X}}_{11}^{\left[j\right]} & \cdots & {\tilde{X}}_{1T}^{\left[j\right]}\\ & \vdots & \\ {\tilde{X}}_{n1}^{\left[j\right]} & \cdots & {\tilde{X}}_{nT}^{\left[j\right]} \end{array}\right]}_{n\times T}\to \;\;{\left[\begin{array}{ccc} \Delta {\tilde{X}}_{11}^{\left[j\right]} & \cdots & \Delta {\tilde{X}}_{1(T-1)}^{\left[j\right]}\\ & \vdots & \\ \Delta {\tilde{X}}_{n1}^{\left[j\right]} & \cdots & \Delta {\tilde{X}}_{n(T-1)}^{\left[j\right]} \end{array}\right]}_{n\times (T-1)},$$

where each $\Delta {\tilde{X}}_{it}^{\left[j\right]}$ is again the first-difference ${\tilde{X}}_{i(t+1)}^{\left[j\right]}-{\tilde{X}}_{it}^{\left[j\right]}$.

To derive the difference design matrix we need to respect the temporality of the observations and regress $\Delta {\tilde{Y}}_{ik}$ for k=1,…,T−1 on the differenced measurements of $\Delta {\tilde{X}}_{it}^{\left[j\right]}$ for t=1,…,k and j=1,…,p. Consequently for the $j{\text{th}}^{\;}$ metabolite the n(T−1)×T(T−1)/2 design matrix is

(5) $$X^{\left[j\right]}=\left[\begin{array}{cccc} \Delta {\tilde{X}}_{11}^{\left[j\right]} & 0 & 0 & 0\\ \vdots & & & \\ \Delta {\tilde{X}}_{n1}^{\left[j\right]} & & & \\ 0 & \Delta {\tilde{X}}_{11}^{\left[j\right]}\;\Delta {\tilde{X}}_{12}^{\left[j\right]} & 0 & 0\\ & \vdots & & \\ & \Delta {\tilde{X}}_{n1}^{\left[j\right]}\;\Delta {\tilde{X}}_{n2}^{\left[j\right]} & & \\ 0 & 0 & \ddots & 0\\ 0 & 0 & 0 & \Delta {\tilde{X}}_{11}^{\left[j\right]}\;\cdots \;\Delta {\tilde{X}}_{1(T-1)}^{\left[j\right]}\\ & & & \vdots \\ & & & \Delta {\tilde{X}}_{n1}^{\left[j\right]}\;\cdots \;\Delta {\tilde{X}}_{n(T-1)}^{\left[j\right]} \end{array}\right].$$

An appropriate univariate test for whether the effect of a metabolite on the outcome *Y* is the Wald test (Wald 1943) for the coefficients from the model $Y=X^{\left[j\right]}\beta^{\left[j\right]}+u$ where *u* is the mean-zero error vector, and

(6) $$\beta^{\left[j\right]}={\left[\beta_{11}^{\left[j\right]}\;\;|\;\beta_{12}^{\left[j\right]}\;\beta_{22}^{\left[j\right]}\;\;|\;\cdots \;\;|\;\beta_{1(T-1)}^{\left[j\right]}\;\cdots \;\beta_{\left(T-1)(T-1\right)}^{\left[j\right]}\right]}^{⊤}.$$

For the test parameters we use the T(T−1)/2 coefficients from the (T−1) models for each component of *Y*, and for the covariance matrix we use a block diagonal with (T−1) blocks containing the covariance matrix for the coefficients of the respective models. This univariate test encapsulates the model structure we are proposing and could be used for small sets of variables that are not heavily correlated. For larger or correlated variable sets we will need a joint model with appropriate constraints.

### 2.3 PROLONG: multivariate case

The above construction gives us the desired regression structure, though we now need to incorporate all metabolites simultaneously for a joint model. The easiest way to do so given our eventual construction of a correlation graph is to replace $\Delta {\tilde{X}}_{ik}^{\left[j\right]}$ in $X^{\left[j\right]}$ above with row vector

(7) $$\Delta {\tilde{X}}_{ik}=\left[\begin{array}{cccc} \Delta {\tilde{X}}_{ik}^{\left[1\right]} & \Delta {\tilde{X}}_{ik}^{\left[2\right]} & \cdots & \Delta {\tilde{X}}_{ik}^{\left[p\right]} \end{array}\right].$$

With these substitutions we get the differenced n(T−1)×pT(T−1)/2 design matrix

(8) $$X=\left[\begin{array}{cccc} \Delta {\tilde{X}}_{11} & 0 & 0 & 0\\ \vdots & & & \\ \Delta {\tilde{X}}_{n1} & & & \\ 0 & \Delta {\tilde{X}}_{11}\;\Delta {\tilde{X}}_{12} & 0 & 0\\ & \vdots & & \\ & \Delta {\tilde{X}}_{n1}\;\Delta {\tilde{X}}_{n2} & & \\ 0 & 0 & \ddots & 0\\ 0 & 0 & 0 & \Delta {\tilde{X}}_{11}\;\cdots \;\Delta {\tilde{X}}_{1(T-1)}\\ & & & \vdots \\ & & & \Delta {\tilde{X}}_{n1}\;\cdots \;\Delta {\tilde{X}}_{n(T-1)}) \end{array}\right].$$

Using the matrix representations of the difference based dependent and independent variables in Equations (3) and (8) the longitudinal regression model takes the form Y=Xβ+u where *u* is the mean-zero error vector and $\beta ={\left[\beta_{11}\;|\;\beta_{12}\;\beta_{22}\;\;|\;\cdots \;|\;\beta_{1(T-1)}\;\cdots \;\beta_{\left(T-1)(T-1\right)}\right]}^{⊤}$ with $\beta_{kl}=\left[\beta_{kl}^{\left[1\right]}\;\beta_{kl}^{\left[2\right]}\;\cdots \;\beta_{kl}^{\left[p\right]}\right]$ for 1≤k≤l≤T−1.

Here, we scale the columns of *X*, but this is a zero intercept model and we do not center the columns of ΔX.

The basic regression model does not contain a known underlying graph connecting the dependence among the independent variables (metabolites), so we now develop a graph-based penalization via the design matrix *X* dependence structure. Since each of the *T−*1 separate time components of *Y* are jointly modeled the block diagonal dependence matrix will mirror our design matrix.

For ith subject, define $\Delta X_{i}$ as the (T−1)×p matrix with the kth (k=1,…,T−1) row equal to Equation (7). Dropping the *i* subscript, define a dependence matrix (such as Pearson or Spearman correlation, or Kendall’s *τ*) of vec(ΔX) to be

(9) $$\tilde{R}={\left[\begin{array}{ccc} {\tilde{R}}^{\left[11\right]} & \cdots & {\tilde{R}}^{\left[1p\right]}\\ \vdots & \ddots & \vdots \\ {\tilde{R}}^{\left[p1\right]} & \cdots & {\tilde{R}}^{\left[pp\right]} \end{array}\right]}_{p(T-1)\times p(T-1)}.$$

Each block ${\tilde{R}}^{\left[j,k\right]}$ is a (T−1)×(T−1) dependence matrix for the columns of X, that is, the block of dependencies among the independent variables.

Now consider the dependence matrix R of $\text{vec}(\Delta X^{⊤})$. This is also a p(T−1)×p(T−1) matrix but can be written with blocks corresponding to pairs of time points

(10) $${\left[\begin{array}{cccc} R_{11} & R_{12} & \cdots & R_{1(T-1)}\\ R_{21} & R_{22} & \cdots & R_{2(T-1)}\\ & & \vdots & \\ R_{(T-1)1} & R_{(T-1)2} & \cdots & R_{\left(T-1)(T-1\right)} \end{array}\right]}_{p(T-1)\times p(T-1)}.$$

The dependence matrix associated with the design matrix *X* in Equation (8) can be constructed using the blocks of R as

(11) $$R={\left[\begin{array}{cccc} R_{11} & 0 & \cdots & 0\\ 0 & R_{\left(1:2)(1:2\right)} & \cdots & 0\\ 0 & 0 & \ddots & 0\\ 0 & \cdots & 0 & R \end{array}\right]}_{pT(T-1)/2\times pT(T-1)/2}.$$

PROLONG uses a regular group lasso penalty along with a network constraint via the Laplacian matrix of the graph associated with *R*. First, estimate *R* via $\hat{R}$. We define this graph *G* as having edges e=(u∼v) between columns *u*, *v* of *X* whose edge-weights are $w(u,v)=|{\hat{R}}_{uv}|.$ The degree of each vertex is $d_{u}=\sum_{v∼u}w(u,v)=\sum_{v∼u}|{\hat{R}}_{uv}|.$

As in Li and Li (2008), we define the normalized Laplacian matrix *L* for graph *G* element-wise as

$$L(u,v)=\{\begin{array}{cc} 1-w(u,v)/d_{u} & \;\text{if}\;u=v\;\text{and}\;d_{u}\neq 0\\ -w(u,v)/\sqrt{d_{u}d_{v}} & \;\text{if}\;u\;\text{and}\;v\;\text{are}\;\text{adjacent}\;\\ 0 & \;\text{otherwise}. \end{array}$$

The penalization criterion used by Li and Li (2008) contains terms for the network constraint and for the lasso penalty, and for our data would be $\lambda_{1}|\beta {|}_{1}+\lambda_{2}\beta^{T}L\beta$.

Following Lemma 1 in Li and Li (2008), given non-negative *λ*_1_, *λ*_2_, we create a new augmented dataset by appending a 0-vector to *Y* and $S^{T}$ to *X*, where $S=\Gamma D^{1/2}$ for $L=\Gamma D\Gamma^{T}$, resulting in (Y,X)
 

$$\begin{array}{ccc} X_{\lambda_{2}}={\left(1+\lambda_{2}\right)}^{-1/2}\left[\begin{array}{c} X\\ \sqrt{\lambda_{2}}S^{T} \end{array}\right], & & Y=\left[\begin{array}{c} Y\\ 0 \end{array}\right] \end{array}.$$

Operationally, we estimate $\beta^{*}$ via lasso on the augmented data then rescale by ${\left(1+\lambda_{2}\right)}^{-1/2}$ to debias.

The group lasso penalty (Yuan and Lin 2006) is defined as $\lambda_{1}\sum_{k=1}^{K}\sqrt{p_{k}}{\left\|\beta^{\left(k\right)}\right\|}_{2}$, for $\lambda_{1}>0$, where ${\left\|\beta^{\left(k\right)}\right\|}_{2}=\sqrt{\sum_{i\in I_{k}}\beta_{i}^{2}}$, for each *k*, the index for group and *p_k_* is the size of group *k*. Put together, the group lasso + Laplacian penalty is

(12) $$\lambda_{1}\sum_{k=1}^{K}\sqrt{p_{k}}{\left\|\beta^{\left(k\right)}\right\|}_{2}+\lambda_{2}\beta^{⊤}L\beta .$$

This penalty is convex as it is the sum of two convex penalties. As in the lasso + Laplacian model, compute S from the $\Gamma D\Gamma^{T}$ decomposition of *L* and construct augmented dataset $\left(Y,X_{\lambda_{2}}\right)$. We apply the group lasso penalty using group-wise-majorization-descent (GMD) (Yang and Zou 2015) implemented in the R package “gglasso” (Yang *et al.* 2020).

(13) $$\underset{\beta }{\mathrm{argmin}}\frac{1}{2}||Y-X_{\lambda_{2}}\beta^{*}|{|}_{2}^{2}+\lambda_{1}\sum_{k=1}^{K}\sqrt{p_{k}}{\left\|\beta^{*}{}^{\left(k\right)}\right\|}_{2}\;\;\lambda_{1},\lambda_{2}>0.$$

The tuning parameter *λ*_2_ is selected via MLE, using the following optimization problem from Steiner *et al.* (2022)
 

(14) $$\begin{array}{c} n\ln\left[{\left\|Y\right\|}_{2}^{2}-Y^{⊤}X_{\lambda_{2}}{\left(B_{\lambda }+X_{\lambda_{2}}^{⊤}X_{\lambda_{2}}\right)}^{-1}X_{\lambda_{2}}^{⊤}Y\right]\\ +\ln\left|B_{\lambda }+X_{\lambda_{2}}^{⊤}X_{\lambda_{2}}\right|-\ln\left|B_{\lambda }\right|, \end{array}$$

where $B_{\lambda }=\lambda_{2}L+\lambda_{R}I.$ The $\lambda_{R}I$ term ensures $B_{\lambda }$ is invertible.

After minimizing over both *λ*_2_ and *λ_R_*, we add $\lambda_{R}I$ to *L* before computing S via $\Gamma D\Gamma^{T}$ decomposition. Then *λ*_1_ is selected via group lasso cross-validation on the augmented model (Y,X) generated using *λ*_2_ and *λ_R_*. Upon solving for $\beta^{*}$, we rescale by ${\left(1+\lambda_{2}\right)}^{-1/2}$.

PROLONG inherits characteristics of both network-constrained regularization and group-lasso. The group lasso penalty ensures that all coefficients associated with a given omics feature are either zero or non-zero, but not necessarily identical. The network constraint guarantees that if *x_i_* = *x_j_* then ${\hat{\beta }}_{i}={\hat{\beta }}_{j}$, and omics features that are highly correlated but not identical will be grouped together. This grouping effect as well as asymptotic results for the group lasso + Laplacian model are described in the following theorems and the proofs are provided in the Supplementary.

Theorem 1. **(Grouping Effect)**. *Given dataset* (Y,X)  *and two fixed scalars* $\left(\lambda_{1},\lambda_{2}\right)$*, the response Y is centered and predictors X are standardized. Let* $\hat{\beta }\left(\lambda_{1},\lambda_{2}\right)$  *be the solution to* $\hat{\beta }={\mathrm{argmin}}_{\beta }\left\{L\left(\lambda_{1},\lambda_{2},\beta \right)\right\}$*. Suppose that* ${\hat{\beta }}_{i}\left(\lambda_{1},\lambda_{2}\right){\hat{\beta }}_{j}\left(\lambda_{1},\lambda_{2}\right)>0$*, the group sizes p_i_ and p_j_ are the same, and the two vertices i and j are only linked to each other on the network*, $d_{i}=d_{j}=w(i,j)$*. Define*
 

$$D_{\lambda_{1},\lambda_{2}}(i,j)=\frac{1}{||Y|{|}_{1}}\left|{\hat{\beta }}_{i}\left(\lambda_{1},\lambda_{2}\right)-{\hat{\beta }}_{j}\left(\lambda_{1},\lambda_{2}\right)\right|.$$

*Then*
 

$$D_{\lambda_{1},\lambda_{2}}(i,j)\leq \frac{1}{2\lambda_{2}}\sqrt{2(1-\rho )}+\frac{\lambda_{1}\sqrt{p_{i}}}{\lambda_{2}||Y|{|}_{1}},$$

*where* $||Y|{|}_{1}=\sum_{i=1}^{n}\left|Y_{i}\right|$  *and* $\rho =X_{.i}^{T}X_{.j}$  *captures the sample correlation for the ith and jth columns of X*, $X_{.i}$*, and* $X_{.j}$.

Theorem 2. **(Asymptotic Property)**. *Let* ${\hat{\beta }}_{n}$  *denote the PROLONG solution for a problem with sample size n, using tuning parameters* $\lambda_{n}^{\left(1\right)}$  *and* $\lambda_{n}^{\left(2\right)}$*. If* $\lambda_{n}^{\left(l\right)}/\sqrt{n}\to \lambda_{0}^{\left(l\right)}\geq 0$  *for l = *1, 2*, and*
 

$$C=\underset{n\to \infty }{\lim}\left(\frac{1}{n}\sum_{i=1}^{n}X_{i.}X_{i.}^{T}\right)$$

*is non-singular, then*
 

$$\sqrt{n}\left({\hat{\beta }}_{n}-\beta \right)\to^{d}\mathrm{argmin}(V),$$

*where*
 

$$\begin{array}{c} V(u)=-2u^{T}W+u^{T}Cu\\ +\lambda_{0}^{\left(1\right)}\sum_{k=1}^{K}\sqrt{p_{k}}(\frac{u^{(k)T}\beta^{\left(k\right)}}{||\beta^{\left(k\right)}|{|}_{2}}I(\beta^{\left(k\right)}\neq 0)\\ +||u^{\left(k\right)}|{|}_{2}I(\beta^{\left(k\right)}=0))\\ +2\lambda_{0}^{\left(2\right)}\sum_{i∼j}\left(\frac{\beta_{i}}{\sqrt{d_{i}}}-\frac{\beta_{j}}{\sqrt{d_{j}}}\right)\left(\frac{u_{i}}{\sqrt{d_{i}}}-\frac{u_{j}}{\sqrt{d_{j}}}\right)w(i,j), \end{array}$$

*and*
 

$$W∼N\left(0,\sigma^{2}C\right).$$

## 3 Results

First, we will compare the performance of PROLONG to that of our benchmark univariate mixed effects models for simulated uncorrelated data with *Y* generated on the first-difference scale. Uncorrelated data is a good first comparison as the network constraint portion of PROLONG will not have any real signal to work with, so it could be thought of as more challenging than data with correlated predictors. Then we will compare PROLONG and benchmarks on correlated data with *Y* generated on the first-difference scale. Finally we will compare PROLONG and benchmarks on correlated data with *Y* generated on the levels scale, both with and without a time-varying intercept *α_t_*.

We are not so much interested in recovering the exact generating coefficients as we are in correctly distinguishing between targets and noise. To assess the performance of PROLONG and the univariate method we will compare the selection rates for each variable, target and noise, across 100 simulations for each scenario.

### 3.1 Simulated differenced-scale data with uncorrelated predictors

The following simulation setup emulates aspects of the real data with varying levels of signal giving an overall signal-to-noise ratio of 1.5. We run small, large, and full data-scale simulations with $p_{\text{targets}}$ = 10, 20, 50 and $p_{\text{noise}}$ = 20, 80, 300, respectively. The data-generating scheme sets

$$y_{1}∼N(15,\sigma^{2}I);\;\sigma^{2}=5p_{\text{targets}}y_{t}∼N(y_{t-1}+\beta (x_{2}-x_{1}),\sigma^{2}I)\;\text{for}\;t\in 2,3,4.x_{1}∼N(\mu ,\Sigma_{X});\;\beta =(\underset{p_{\text{targets}}}{\underset{︸}{1,\ldots ,1}},\underset{p_{\text{noise}}}{\underset{︸}{0,\ldots ,0}})\mu ∼U(10,20),\Sigma_{X}=\text{diag}(\sigma_{1},\ldots ,\sigma_{p}),\sigma_{j}∼U(1,2);x_{2}∼x_{1}+N(d\mu ,\Sigma_{X});\;d\mu =(\underset{p_{\text{targets}}}{\underset{︸}{5,\ldots ,10}},\underset{p_{\text{noise}}}{\underset{︸}{0,\ldots ,0}});x_{t}∼x_{t-1}+N(0,\Sigma_{X})\;\text{for}\;t\in 3,4.$$

#### 3.1.1 Small scale results

**Figure 2.**
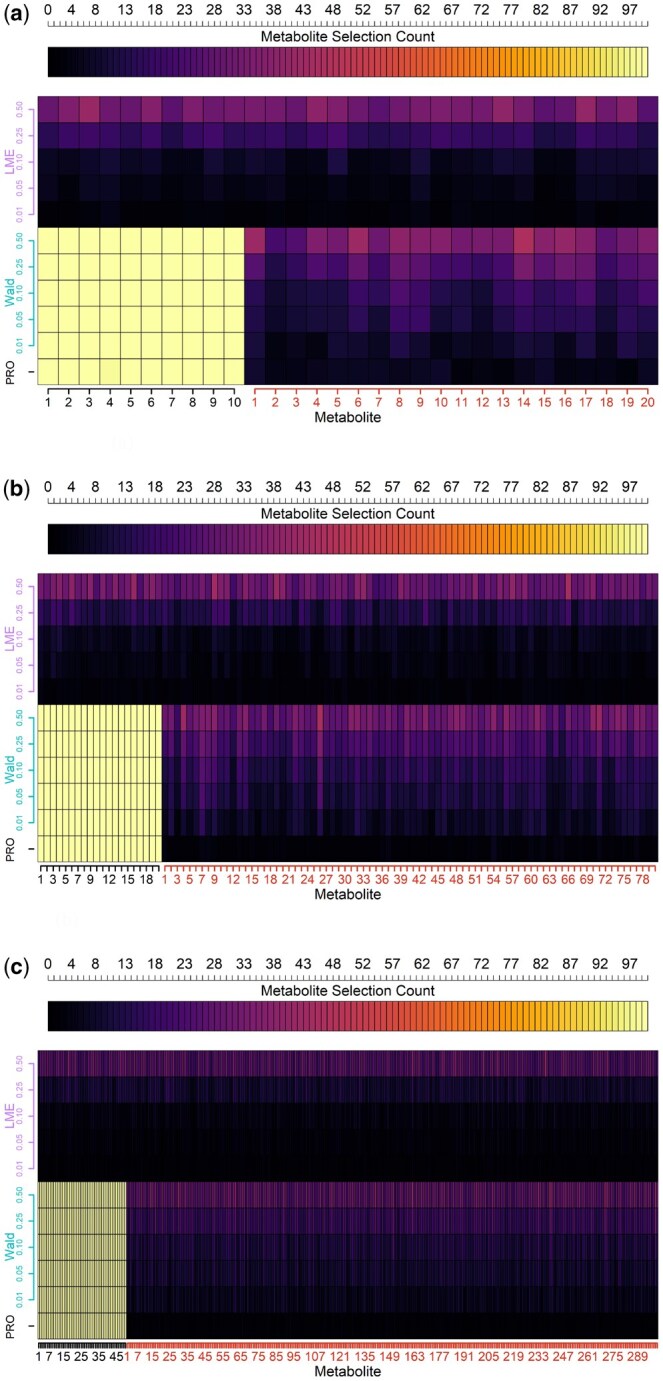
Comparison of selection counts (top axis) across simulations for target and noise metabolites (*x*-axis) using PROLONG, univariate Wald tests, and univariate Linear Mixed Effects (LME) models at Various FDR Thresholds (*y*-axis). PROLONG and its Wald analog both have perfect or near-perfect sensitivity across scenarios and FDR thresholds, PROLONG has higher specificity across the scenarios than Wald FDR 0.05, and the linear mixed effects models struggle everywhere. (a) Simulated uncorrelated data, 10 targets and 20 noise. (b) Simulated uncorrelated data, with 20 target and 80 noise variables. (c) Simulated uncorrelated data, 50 targets and 300 noise.

As seen in Fig. 2a, with $p_{\text{targets}}=10$ and $p_{\text{noise}}=20$, we find that the univariate mixed effects models struggled to distinguish between targets and noise. This pattern holds across FDR thresholds, as the rate of acceptance increased flatly across targets and noise. PROLONG picked up 99.2% of the targets and 3.75% of the noise across simulations. At an FDR of 0.05, the Wald test selected every target and 11.15% of the noise across simulations.

Figure S6 in the Supplementary File shows the coefficient paths and group lasso CV loss for a simulation run from each of the correlated and uncorrelated small-scale simulations. The blue lines show the coefficient paths for the target variables, red show the paths for the noise variables. The higher blue paths indicate coefficients for the time points that are used to generate the changes in *Y*, while those closer to zero represent time points not used to generate *Y* but that are kept from being shrunk to zero by the group penalty. In this example of the uncorrelated data, we see a wide gap in the log lambda scale for the selection of noise and targets. We can observe a steady decline in the noise coefficients, while the non-significant time points for the target variables are relatively flat across the lambda scale.

#### 3.1.2 Large scale results

Figure 2b shows that when $p_{\text{targets}}=20$ and $p_{\text{noise}}=80$ PROLONG selected every target metabolite and 0.43% of the noise. The Wald tests also selected the targets perfectly at an FDR of 0.05 along with 9.9% of the noise. With this larger variable set we see a much stronger performance for PROLONG compared to the Wald test. Again, the univariate mixed effects models struggled as expected with flat selection across noise and target variables.

#### 3.1.3 Full data scale results

Figure 2c shows results for the full data-scale results where $p_{\text{targets}}=50$ and $p_{\text{noise}}=300$. The univariate mixed effects models again showed no difference across target and noise. PROLONG and Wald at an FDR of 0.05 both select all targets once again, with 0.18% and 8.8% of noise picked up, respectively.

### 3.2 Simulated differenced-scale data with correlated predictors

Next, we will compare PROLONG and the univariate tests for performance on simulated correlated data. The univariate mixed effects models cannot make use of any correlation between variables, so we do not expect the results to vary meaningfully between uncorrelated and correlated simulations.

This simulation setup only differs from the previous by the definition of Σ_*X*_. Here we have $\Sigma_{X}=\left[\begin{array}{cc} \Sigma_{C} & 0\\ 0 & \Sigma_{\epsilon } \end{array}\right]$ where for $\Sigma_{d}=\text{diag}(\sigma_{1},\ldots ,\sigma_{p})$ our covariance matrix $\Sigma_{C}=\Sigma_{d}C\Sigma_{d}$ associated with our target variables is constructed so that the variances on the diagonal are in the same range as those of the noise variables, while the off-diagonal elements correspond to off-diagonal elements of correlation matrix *C* drawn uniformly from (−1,1).

Constructing Σ_*X*_ in this way ensures we have some (random) correlation between the target variables that generate *Y* that can be leveraged by the correlation network penalty.

#### 3.2.1 Small scale results

Figure S1a in the Supplementary File displays our results when $p_{\text{targets}}=10$ and $p_{\text{noise}}=20$. The univariate mixed effects model struggled as in the uncorrelated case, while PROLONG picked up 95% of targets and Wald tests picked up every target. PROLONG selected only 5.75% of the noise, lower than Wald tests at an FDR of 0.05 with 11.05%.

#### 3.2.2 Large scale results

Figure S1b in the Supplementary File shows results for $p_{\text{targets}}=20$ and $p_{\text{noise}}=80$. PROLONG and Wald tests at an FDR of 0.05 selected all of the targets along with 0.62% and 9.8%, respectively.

#### 3.2.3 Full data scale results

Figure S1c in the Supplementary File shows results for the full data-scale results where $p_{\text{targets}}=50$ and $p_{\text{noise}}=300$. The univariate mixed effects models again showed no difference across target and noise. PROLONG and Wald tests at an FDR of 0.05 both select all targets once again along with 0.18% and 8.74% of the noise, respectively.

### 3.3 Simulated levels-scale data with correlated predictors

We will now compare PROLONG and the univariate tests on simulated correlated data where the outcome is generated from the target variables on the levels-scale rather than the first-differenced scale. The simulation setup is shown below, using the same *μ* and Σ_*X*_ as in the first differenced-scale correlated setup. For brevity we fix $p_{\text{targets}}=20$ and $p_{\text{noise}}=80$, and look at different values for the time intercept *α_t_*. This scheme sets

$$y_{t}∼N(\alpha_{t}+\beta x_{t},\sigma^{2}I);\;\sigma^{2}=5;\;\alpha_{t}\in (0,t,5t)x_{1}∼N(\mu ,\Sigma_{X});\;\beta =(\underset{20}{\underset{︸}{5,\ldots ,5}},\underset{80}{\underset{︸}{0,\ldots ,0}})/p_{\text{targets}}x_{2}∼x_{1}+N(d\mu_{1},\Sigma_{X});\;d\mu_{1}=(\underset{20}{\underset{︸}{10,\ldots ,10}},\underset{80}{\underset{︸}{0,\ldots ,0}});x_{t}∼x_{t-1}+N(d\mu_{2},\Sigma_{X});\;t\in 3,4;d\mu_{2}=(\underset{10}{\underset{︸}{0,\ldots 0,}}\underset{10}{\underset{︸}{-5,\ldots ,-5}},\underset{80}{\underset{︸}{0,\ldots ,0}}).$$

#### 3.3.1 No time-varying intercept

Figure S2a in the Supplementary File shows the results with *α_t_* = 0. PROLONG selects all targets and 5.7% of the noise across simulations. At an FDR of 0.05, the Wald tests and LMEs models selected 100% and 90.7% of the targets, respectively, along with 9.075% and 4.2% of the noise.

#### 3.3.2 With time-varying intercept

Figure S2b in the Supplementary File shows the results with *α_t_* = *t*. PROLONG again selects all targets and slightly more noise at 6%. The Wald tests select all targets and 8.5% of the noise, and the LMEs models pick 89.4% of the targets and, as with $\alpha_{t}=0$, 4.2% of the noise.

#### 3.3.3 With large time-varying intercept

Figure S2c in the Supplementary File shows the results with *α_t_* = 5*t*. PROLONG still selects all targets, along with a higher 16% of the noise. The Wald tests select all targets with 7.6% of the noise, and the LMEs models match the $\alpha_{t}=t$ scenario with 89% of targets and 4.2% of noise.

### 3.4 Summary of simulation results

For the first difference-scale simulations we can observe that PROLONG had near-perfect sensitivity in both correlated and uncorrelated simulated data and improved as we increased our variable size and sparsity. The univariate mixed effects models struggled in all scenarios. The Wald tests at an FDR threshold of 0.05 picked up every target, but also much more noise than PROLONG and improved more slowly as dimension increased. The correlation did not provide a significant advantage here. Even uncorrelated variables with a lower signal were picked up nearly always across all scenarios, so there was little room for improvement with the incorporation of a correlation structure within the target variables.

The levels-scale simulations show much better success for the LMEs models, while still preserving a strong performance for PROLONG and the Wald tests. The LME models miss about 10% of the targets but have a flat noise rate of 4.2%. The Wald tests pick up every target but have higher noise across *α_t_*. In the large *α_t_* scenario PROLONG selects more noise than either univariate model, but in the other scenarios it is slightly above the LME models and below Wald tests. PROLONG also selects every target across scenarios. It should be noted that in this final scenario $\alpha_{t}=\sum \beta$. Even in the presence of this very large time intercept PROLONG preserves its sensitivity, only picking up additional noise to fill in the *α_t_* signal.

Additional results are shown in the Supplementary File. Figure S3 shows various scenarios as before (delta- and levels-scale) with an additional levels-scale version of the PROLONG model (denoted LEV) included. This levels-scale version of PROLONG has much less power than the delta-scale PROLONG across all simulations. Figure S4 shows the same scenarios and models as Fig. S3 but with *X* generated from cubic splines. Levels-scale PROLONG struggles even more in these scenarios, while PROLONG only slightly loses power. Figure S5 shows scenarios comparing PROLONG, its levels-scale version, and PGEE (Wang *et al.* 2012). PGEE fails to distinguish between targets and noise, with low overall power as well.

### 3.5 Data

#### 3.5.1 Clinical significance

TB remains a major global health issue, with an estimated 10 million new cases and 1.4 million deaths annually, despite being preventable and treatable (WHO *et al.* 2021). Challenges include long treatment durations, rising drug resistance, and a lack of reliable biomarkers for diagnosis and for monitoring treatment efficacy (Das *et al.* 2016, Dartois and Rubin 2022, Salari *et al.* 2023, Gillespie *et al.* 2024). We are investigating the potential of urinary metabolomic data from TB trials as biomarkers for early bactericidal activity.

While immediate motivation for the proposed method stems from the TB study, the development of statistical models for short-panel omics data may enhance our ability to handle similar data structures in diverse medical research. In fact, this data is reflective of an increasingly common collection of biomarkers across a few time points with some continuous outcome of interest. There is extensive work on differential expression methods, but here we have monotonically increasing but variable improvement in TTP across subjects, as well as potentially interesting biomarkers that are not significantly different from the first and last time points of measurement. While some metabolites do show a strong spike and plateau pattern, others primarily show variability between subjects at the first few time points while having a relatively stable mean. This data is representative of other new datasets while also providing a challenge that existing methods are not intended to address.

#### 3.5.2 Collection and preprocessing

Missingness is a common issue in mass-spectometry based metabolomics data (Wei *et al.* 2018), and so for the purpose of model development we limited the following analysis to metabolites with less than 20% observations missing across subjects and time points. We have 352 metabolites that meet this criterion, of which 79 have no missing values. We split the mostly complete 352 metabolites into 4 matrices, one for each time point, and used softImpute (Mazumder *et al.* 2010, Hastie and Mazumder 2021) to fill in missing values for each time point’s matrix of metabolite abundances by subject. Imputation was done in this way to avoid introducing any new dependence across time points.

#### 3.5.3 Benchmark method

As in the simulations we are interested in the model results as classifying targets versus noise. We investigate the selected metabolites for PROLONG, our Wald tests, and the univariate longitudinal mixed effects models, checking for target metabolites identified in our exploratory analysis and previous literature.

#### 3.5.4 Data results

No metabolites are selected by the univariate mixed effects models. This approach likely struggles with the apparent differential nature of the data. The data result mirror the first difference-scale simulations where Wald and PROLONG select many but the mixed effects models select very few amongst targets or noise.

**Figure 3.**
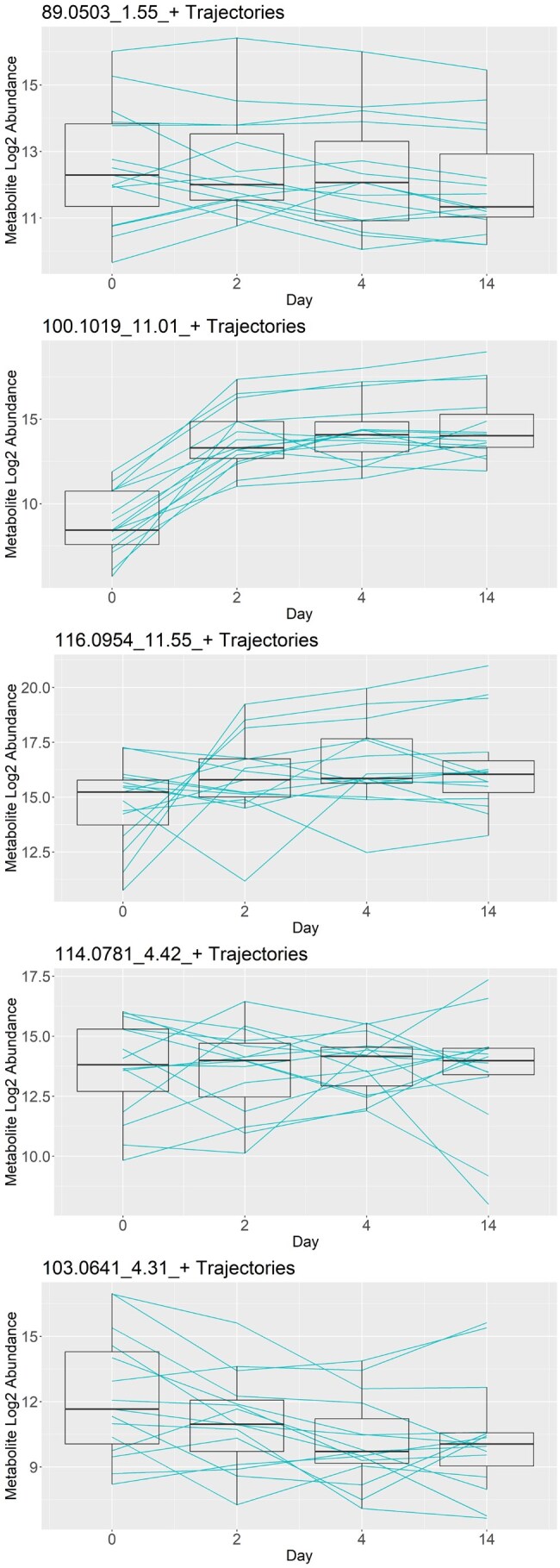
Trajectories for the five metabolites selected by PROLONG with the highest coefficients, excluding 204.1867_9.3_+ shown in the introduction. These metabolites show a variety in trend and variance over time and demonstrate the versatility of PROLONG.

Figure 3 shows 5 of the 29 metabolites selected by the PROLONG model, sorted by average coefficient magnitude but excluding 204.1867_9.3_+ shown in the introduction. PROLONG also picks up 344.0923_0.01_- shown in Fig. 1. 100.1019_11.01_+ shows a pattern similar to 204.1867_9.3_+, but among the rest we don’t always have the same clear spike and plateau. There are still easily observable differential effects in at least a few subjects that were picked up by the model. We can also observe that PROLONG does not only pick up metabolites that are differentially expressed between the first and last time points.

## 4 Discussion

Data and figures shown in this paper illustrate the effectiveness of PROLONG as well as its univariate Wald test analog to a lesser extent. PROLONG can be thought of as a principled classification method where we are not so much interested in the coefficients themselves or least squares error as we are in statistical power. The simulations show that both sensitivity and specificity are very high, particularly in the larger simulated datasets, and the real metabolites selected by PROLONG show the type of biological signal that motivated the model itself. Notably, PROLONG does not only select those metabolites that are strongly differentially expressed before and after treatment but also those that are relatively consistent in mean but show subject variability patterns that correspond to subject variability in improvement in TTP over time.

Using difference-scale time-varying coefficients for our variables does not allow for these coefficients to be identifiable with the inclusion of an intercept term. However, we do not have indication that this should pose a significant problem- the additional noise selected is minimal with reasonable *α_t_*’s. The simulations indicate that PROLONG selects significantly less noise than its Wald test analog when *α_t_* is zero or reasonably small, and that the LMEs models struggle on the first-differenced scale. If our data had a large *α_t_* we would expect more selection from PROLONG than from the Wald test analog, and if the Y∼X relationship was better explained on the levels-scale than the first-difference scale we would expect some selections from the LMEs models. The use of PROLONG for this data allows for the identification of metabolites that would have been missed with LMEs models.

There is limited preprocessing necessary for PROLONG; all that is necessary is first-differencing the data, scaling the predictors, and computing the incidence matrix. Prior biological knowledge is helpful, and could be incorporated into the network penalty in place of absolute correlation in appropriate scenarios. By default, however, the network penalty uses observed correlations rather than known pathways and can be used as-is for data without a known connective structure or for data where molecule identification is possible but costly.

The extension of PROLONG to multiple types of continuous omics data is immediate. The graph and model assumptions do not require a specific covariance structure, so we could combine the multiple omics types column-wise before proceeding with the same design matrix stacking and model procedure. For the graph edge weights, the correlation between two individual omics of different types is already interpretable, so we don’t expect to need any adjustments for this part of the extension. For a mixture of binary, count, and/or continuous omic data types, caution will need to be exercised in the selection of correlation measure for the graph’s edge weights. This method extension provides a clear path towards effective multi-omic integration, not only allowing for simultaneous feature selection but also incorporating additional knowledge about how the features co-vary.

The standard group lasso penalty used in PROLONG guarantees all non-zero or all zero coefficients within a group. There may be some advantages to inducing sparsity within each of these groups with sparse group lasso. In many datasets we should expect some time points, such as those closest to treatment, will be more important than others. Within-group sparsity may reveal additional information about the temporal relationship between the outcome and selected variables in such data.

The inclusion of continuous demographic or other time-invariant variables can be accomplished by adding an additional *Zb* term. This term would allow cross-sectional variables to be included outside the network and group lasso constraints.

## Supplementary Material

btaf099_Supplementary_Data

## Data Availability

The data underlying this article cannot be shared publicly to maintain the privacy of the individuals that participated in the study. The data will be shared on reasonable request to the corresponding author.
