# Supplementary material for: PROLONG: penalized regression for outcome guided longitudinal omics analysis with network and group constraints
Source: Bioinformatics. 2025 Mar 7;41(4):btaf099. doi: 10.1093/bioinformatics/btaf099 (PMC11955234; doi:10.1093/bioinformatics/btaf099)
Supplement: btaf099_Supplementary_Data [file btaf099_supplementary_data.pdf]

# 1 Theorems

**Theorem 1.** ***(Grouping Effect).*** *Given dataset $(Y, X)$ and two fixed scalars $(\lambda_1, \lambda_2)$, the response $Y$ is centered and predictors $X$ are standardized. Let $\hat{\beta}(\lambda_1, \lambda_2)$ be the solution to $\hat{\beta} = \mathrm{argmin}_\beta \{L(\lambda_1, \lambda_2, \beta)\}$. Suppose that $\hat{\beta}_i(\lambda_1, \lambda_2)\hat{\beta}_j(\lambda_1, \lambda_2) > 0$, the group sizes $p_i$ and $p_j$ are the same, and the two vertices $i$ and $j$ are only linked to each other on the network, $d_i = d_j = w(i, j)$. Define*

$$D_{\lambda_1,\lambda_2}(i,j) = \frac{1}{||Y||_1}\left|\hat{\beta}_i(\lambda_1, \lambda_2) - \hat{\beta}_j(\lambda_1, \lambda_2)\right|.$$

*Then*

$$D_{\lambda_1,\lambda_2}(i,j) \leq \frac{1}{2\lambda_2}\sqrt{2(1-\rho)} + \frac{\lambda_1\sqrt{p_i}}{\lambda_2||Y||_1},$$

*where $||Y||_1 = \sum_{i=1}^{n}|Y_i|$ and $\rho = X_{.i}^T X_{.j}$ captures the sample correlation.*

*Proof.* Because $\hat{\beta} = \mathrm{argmin}_\beta \{L(\lambda_1, \lambda_2, \beta)\}$, it satisfies

$$\left.\frac{\partial L(\lambda_1, \lambda_2, \beta)}{\partial \beta_k}\right|_{\beta=\hat{\beta}(\lambda_1,\lambda_2)} = 0 \quad \text{if} \quad \hat{\beta}_k(\lambda_1, \lambda_2) \neq 0.$$

When $p_k = 1\ \forall k$, then the group lasso penalty becomes the lasso, and the proof comes from (Li and Li 2008). We present it here for sake of completeness. The KKT condition implies, for any $i, j \in \{1, \ldots, p\}$, that

$$-2X_{.i}^T\{Y - X\hat{\beta}\} + \lambda_1 \,\mathrm{sgn}\left\{\hat{\beta}_i\right\} + 2\lambda_2\hat{\beta}_i - 2\lambda_2\sum_{u\sim i} w(u,i)\frac{\hat{\beta}_u}{\sqrt{d_u d_i}} = 0,$$

$$-2X_{.j}^T\{Y - X\hat{\beta}\} + \lambda_1 \,\mathrm{sgn}\left\{\hat{\beta}_j\right\} + 2\lambda_2\hat{\beta}_j - 2\lambda_2\sum_{v\sim j} w(v,j)\frac{\hat{\beta}_v}{\sqrt{d_v d_j}} = 0.$$

Taking the difference of the above two equations, we have

$$\left(X_{.j}^T - X_{.i}^T\right)(Y - X\hat{\beta}) + \lambda_1\left(\mathrm{sgn}\left\{\hat{\beta}_i\right\} - \mathrm{sgn}\left\{\hat{\beta}_j\right\}\right) + \lambda_2\left(\hat{\beta}_i - \hat{\beta}_j\right) + \lambda_2\left\{\frac{1}{\sqrt{d_j}}\sum_{v\sim j} w(v,j)\frac{\hat{\beta}_v}{\sqrt{d_v}} - \frac{1}{\sqrt{d_i}}\sum_{u\sim i} w(u,i)\frac{\hat{\beta}_u}{\sqrt{d_u}}\right\} = 0.$$

By assumption, $\mathrm{sgn}\left\{\hat{\beta}_i\right\} = \mathrm{sgn}\left\{\hat{\beta}_j\right\}$ and $d_i = d_j = w(i,j)$, we have

$$\frac{1}{||Y||_1}\left|\left(\hat{\beta}_i - \hat{\beta}_j\right)\right| = \frac{|(X_{.j}^T - X_{.i}^T)||(Y - X\hat{\beta})\,|}{2\lambda_2||Y||_1}.$$

(Zou and Hastie 2005) showed that

$$\frac{|(X_{.j}^T - X_{.i}^T)||(Y - X\hat{\beta})|}{\lambda_2||Y||_1} \leq \frac{1}{\lambda_2}\sqrt{2(1-\rho)}.$$

This completes the proof for $p_k = 1$.
When the group size $p_k > 1$, we have

$$-2X_{.i}^T\{Y - X\hat{\beta}\} + \lambda_1\sqrt{p_i}\frac{\hat{\beta}_i}{||\hat{\beta}^{(k_i)}||_2} + 2\lambda_2\hat{\beta}_i - 2\lambda_2\sum_{u\sim i} w(u,i)\frac{\hat{\beta}_u}{\sqrt{d_u d_i}} = 0,$$

$$-2X_{.j}^T\{Y - X\hat{\beta}\} + \lambda_1\sqrt{p_j}\frac{\hat{\beta}_j}{||\hat{\beta}^{(k_j)}||_2} + 2\lambda_2\hat{\beta}_j - 2\lambda_2\sum_{v\sim j} w(v,j)\frac{\hat{\beta}_v}{\sqrt{d_v d_j}} = 0.$$

Where $k_i$ and $k_j$ represent the groups containing $\beta_i, \beta_j$. Taking the difference of the above two equations gives

$$\left(X_{.j}^T - X_{i.}^T\right)(Y - X\hat{\beta}) + \lambda_1 \left(\sqrt{p_i}\frac{\hat{\beta}_i}{||\hat{\beta}^{(k_i)}||_2} - \sqrt{p_j}\frac{\hat{\beta}_j}{||\hat{\beta}^{(k_j)}||_2}\right) + \lambda_2\left(\hat{\beta}_i - \hat{\beta}_j\right)$$
$$+ \lambda_2 \left\{\frac{1}{\sqrt{d_j}}\sum_{v\sim j} w(v,j)\frac{\hat{\beta}_v}{\sqrt{d_v}} - \frac{1}{\sqrt{d_i}}\sum_{u\sim i} w(u,i)\frac{\hat{\beta}_u}{\sqrt{d_u}}\right\} = 0.$$

By assumption $\sqrt{p_i} = \sqrt{p_j}$

$$\left(X_{.j}^T - X_{.i}^T\right)(Y - X\hat{\beta}) + \lambda_1\sqrt{p_i} \left(\frac{\hat{\beta}_i}{||\hat{\beta}^{(k_i)}||_2} - \frac{\hat{\beta}_j}{||\hat{\beta}^{(k_j)}||_2}\right) + \lambda_2\left(\hat{\beta}_i - \hat{\beta}_j\right)$$
$$+ \lambda_2 \left\{\frac{1}{\sqrt{d_j}}\sum_{v\sim j} w(v,j)\frac{\hat{\beta}_v}{\sqrt{d_v}} - \frac{1}{\sqrt{d_i}}\sum_{u\sim i} w(u,i)\frac{\hat{\beta}_u}{\sqrt{d_u}}\right\} = 0.$$

By assumption, $d_i = d_j = w(i,j)$, so

$$\hat{\beta}_i - \hat{\beta}_j = \frac{1}{2\lambda_2}(X_{.i}^T - X_{.j}^T)\hat{r} - \frac{\lambda_1\sqrt{p_i}}{\lambda_2}(\frac{\hat{\beta}_i}{||\hat{\beta}^{(k_i)}||_2} - \frac{\hat{\beta}_j}{||\hat{\beta}^{(k_j)}||_2}).$$

Where $\hat{r} = Y - X\hat{\beta}$. Because $\hat{\beta} = \text{argmin}_\beta\{L(\lambda_1, \lambda_2, \beta)\}$, then

$$L\left(\lambda_1, \lambda_2, \hat{\beta}\right) \leq L(\lambda_1, \lambda_2, \beta = 0)$$
$$\rightarrow ||\hat{r}||_1^2 \leq ||Y||_1^2.$$

So we have

$$D_{\lambda_1,\lambda_2}(i,j) \leq \frac{1}{2\lambda_2}\frac{||\hat{r}||_1}{||Y||_1} + |\frac{\lambda_1\sqrt{p_i}}{||Y||_1 2\lambda_2}(\frac{\hat{\beta}_i}{||\hat{\beta}^{(k_i)}||_2} - \frac{\hat{\beta}_j}{||\hat{\beta}^{(k_j)}||_2})|$$
$$\leq \frac{1}{2\lambda_2}\sqrt{2(1-\rho)} + \frac{\lambda_1\sqrt{p_i}}{\lambda_2||Y||_1}.$$

□

**Theorem 2.** ***(Asymptotic Property)*** *Let $\hat{\beta}_n$ denote the PROLONG solution for a problem with sample size n, using tuning parameters $\lambda_n^{(1)}$ and $\lambda_n^{(2)}$. If $\lambda_n^{(l)}/\sqrt{n} \rightarrow \lambda_0^{(l)} \geq 0$ for $l = 1, 2$ and*

$$C = \lim_{n\rightarrow\infty}\left(\frac{1}{n}\sum_{i=1}^{n} X_{i.}X_{i.}^T\right)$$

*is non-singular, then*

$$\sqrt{n}\left(\hat{\beta}_n - \beta\right) \rightarrow^d \text{argmin}(V),$$

*where*

$$V(u) = -2u^TW + u^TCu$$
$$+ \lambda_0^{(1)}\sum_{k=1}^{K}\sqrt{p_k}\left(\frac{u^{(k)T}\beta^{(k)}}{||\beta^{(k)}||_2}I\left(\beta^{(k)} \neq 0\right) + ||u^{(k)}||_2 I\left(\beta^{(k)} = 0\right)\right)$$
$$+ 2\lambda_0^{(2)}\sum_{i\sim j}\left(\frac{\beta_i}{\sqrt{d_i}} - \frac{\beta_j}{\sqrt{d_j}}\right)\left(\frac{u_i}{\sqrt{d_i}} - \frac{u_j}{\sqrt{d_j}}\right)w(i,j),$$

*and*

$$W \sim N\left(0, \sigma^2 C\right).$$

*Proof.* This proof is similar to those of the asymptotic results for fused lasso (Tibshirani et al. 2005) and the lasso + Laplacian model (Li and Li 2008) respectively. Define

$$V_n(u) = \sum_{i=1}^{n}\left\{\left(\varepsilon_i - u^T X_{i.}/\sqrt{n}\right)^2 - \varepsilon_i^2\right\} + \lambda_n^{(1)} \sum_{k=1}^{K} \sqrt{p_k}\left(||\beta^{(k)} + u^{(k)}/\sqrt{n}||_2 - ||\beta^{(k)}||_2\right)$$
$$+\lambda_n^{(2)} \sum_{i\sim j}\left\{\left(\left(\frac{\beta_i}{\sqrt{d_i}} - \frac{\beta_j}{\sqrt{d_j}}\right) + \left(\frac{u_i}{\sqrt{d_i}} - \frac{u_j}{\sqrt{d_j}}\right)/\sqrt{n}\right)^2 w(i,j) - \left(\frac{\beta_i}{\sqrt{d_i}} - \frac{\beta_j}{\sqrt{d_j}}\right)^2 w(i,j)\right\}.$$

$V_n(u)$ is minimized at $\sqrt{n}\left(\hat{\beta}_n - \beta\right)$. We can observe that

$$\sum_{i=1}^{n}\left\{\left(\varepsilon_i - u^T X_{i.}/\sqrt{n}\right)^2 - \varepsilon_i^2\right\} \to^d -2u^T W + u^T C u,$$

where as in (Fu and Knight 2000), the finite dimensional convergence holds trivially.
We can also observe, as in (Nardi and Rinaldo 2008), that

$$\lambda_n^{(1)} \sum_{k=1}^{K} \sqrt{p_k}\left(||\beta^{(k)} + u^{(k)}/\sqrt{n}||_2 - ||\beta^{(k)}||_2\right) \to^d \lambda_0^{(1)} \sum_{k=1}^{K} \sqrt{p_k}\left(\frac{u^{(k)T}\beta^{(k)}}{||\beta^{(k)}||_2} I\left(\beta^{(k)} \neq 0\right) + ||u^{(k)}||_2 I\left(\beta^{(k)} = 0\right)\right),$$

and as in (Li and Li 2008) that

$$\lambda_n^{(2)} \sum_{i\sim j}\left\{\left(\left(\frac{\beta_i}{\sqrt{d_i}} - \frac{\beta_j}{\sqrt{d_j}}\right) + \left(\frac{u_i}{\sqrt{d_i}} - \frac{u_j}{\sqrt{d_j}}\right)/\sqrt{n}\right)^2 w(i,j) - \left(\frac{\beta_i}{\sqrt{d_i}} - \frac{\beta_j}{\sqrt{d_j}}\right)^2 w(i,j)\right\}$$
$$\to^d 2\lambda_0^{(2)} \sum_{i\sim j}\left(\frac{\beta_i}{\sqrt{d_i}} - \frac{\beta_j}{\sqrt{d_j}}\right)\left(\frac{u_i}{\sqrt{d_i}} - \frac{u_j}{\sqrt{d_j}}\right) w(i,j).$$

So with $V_n(u) \xrightarrow{d} V(u)$, $V_n$ being convex and $V$ having a unique minimum then from (Geyer 1996) we have $\text{argmin}(V_n) = \sqrt{n}(\hat{\beta}_n - \beta) \xrightarrow{d} \text{argmin}(V)$.

□

# 2 Supplementary Figures

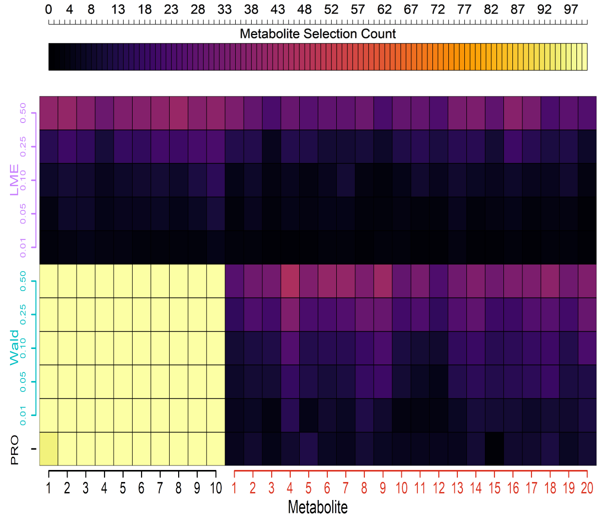


(a) Simulated correlated data, 10 targets and 20 noise

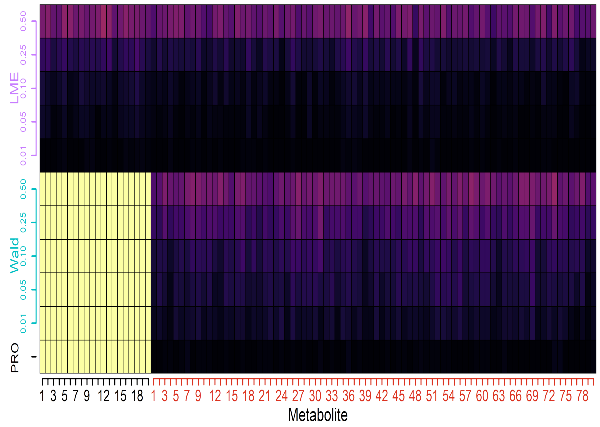


(b) Simulated correlated data, 20 targets and 80 noise

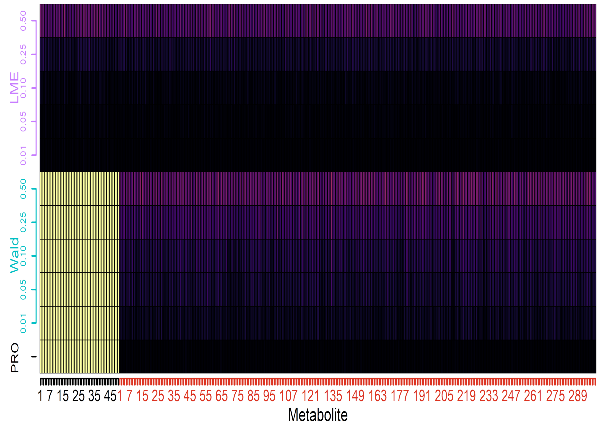


(c) Simulated correlated data, 50 targets and 300 noise

Figure S1: Comparison of selection counts (top axis) across simulations for target and noise metabolites (x-axis) using PROLONG, univariate Wald tests, and univariate Linear Mixed Effects (LME) models at Various FDR Thresholds (y-axis). PROLONG and its Wald analog both have perfect or near-perfect sensitivity across scenarios and FDR thresholds, PROLONG has higher specificity across the scenarios than Wald FDR 0.05, and the linear mixed effects models struggle everywhere.

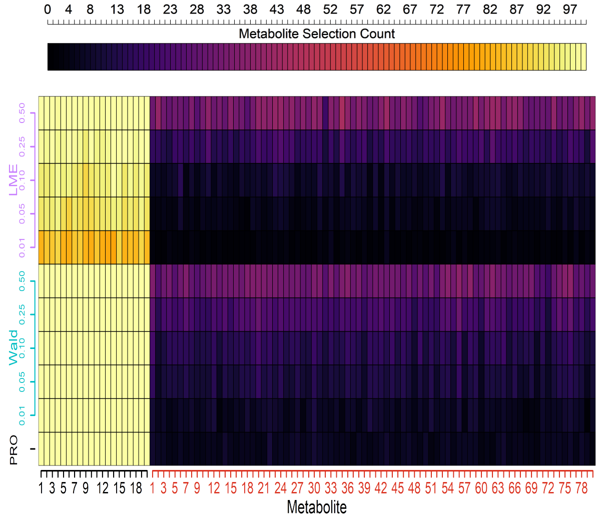


(a) Simulated levels-scale correlated data with no time intercept, 20 targets and 80 noise

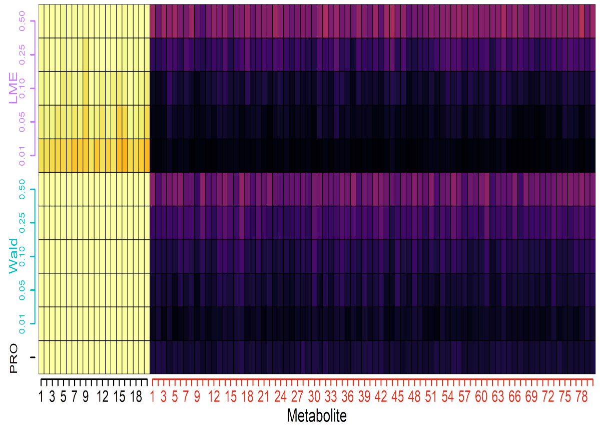


(b) Simulated levels-scale correlated data with time intercept, 20 targets and 80 noise

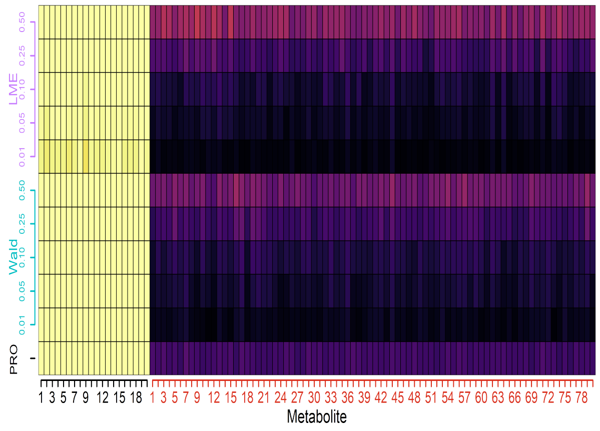


(c) Simulated levels-scale correlated data with large time intercept, 20 targets and 80 noise

Figure S2: Comparison of selection counts (top axis) across simulations for target and noise metabolites (x-axis) using PROLONG, univariate Wald tests, and univariate Linear Mixed Effects (LME) models at Various FDR Thresholds (y-axis). The linear mixed effects models now have high sensitivity, though still lower than Wald and PROLONG at each $\alpha_t$ magnitude. PROLONG performs the best overall for $\alpha_t \in 0, t$ and picks more noise than Wald FDR 0.05 and LME when $\alpha_t = 5t$ is very large while maintaining perfect sensitivity.

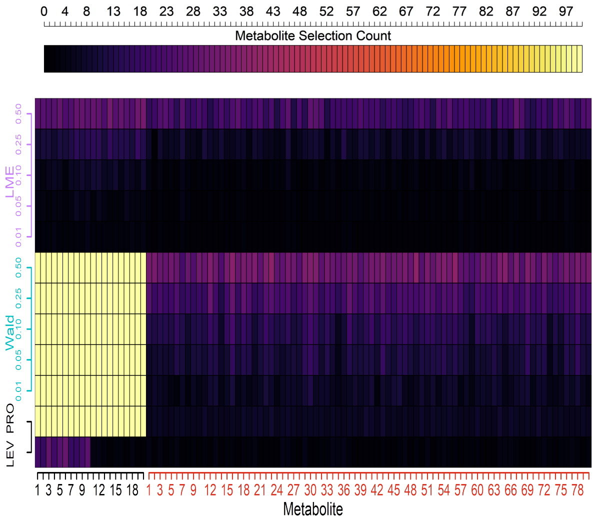


(a) Simulated delta-scale correlated data with no lags, 20 targets and 80 noise

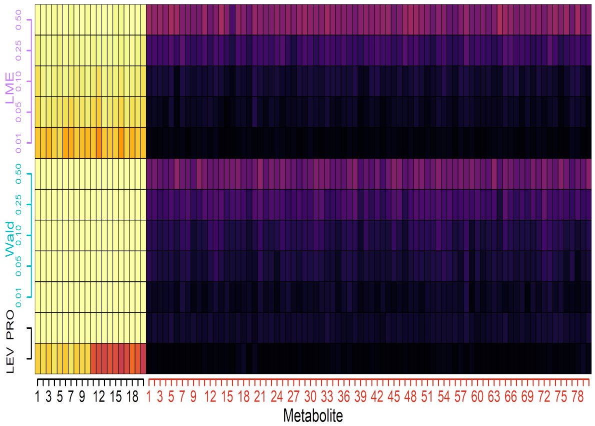


(b) Simulated levels-scale correlated data, 20 targets and 80 noise

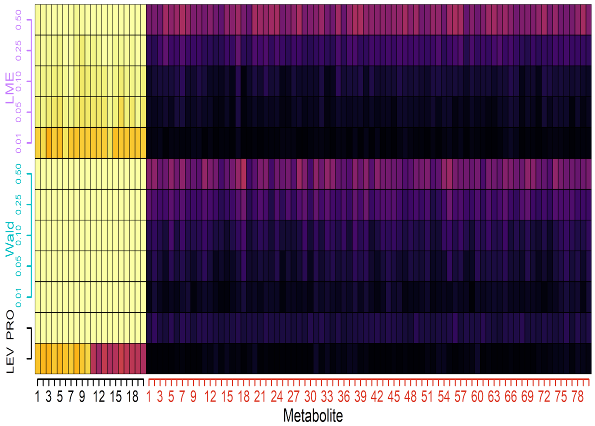


(c) Simulated levels-scale correlated data with time intercept, 20 targets and 80 noise

Figure S3: Comparison of selection counts (top axis) across simulations for target and noise metabolites (x-axis) using PROLONG, a levels scale version of PROLONG (LEV), univariate Wald tests, and univariate Linear Mixed Effects (LME) models at Various FDR Thresholds (y-axis). The levels-scale PROLONG (LEV) is significantly worse than PROLONG at identifying targets in all scenarios.

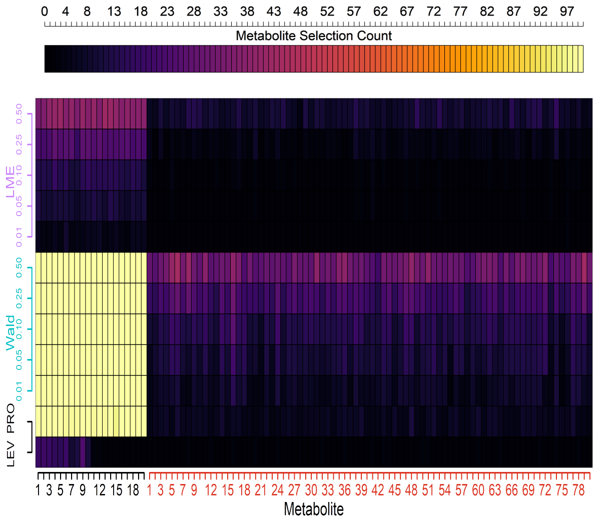


(a) Y generated on delta-scale from cubic spline data with no lags, 20 targets and 80 noise

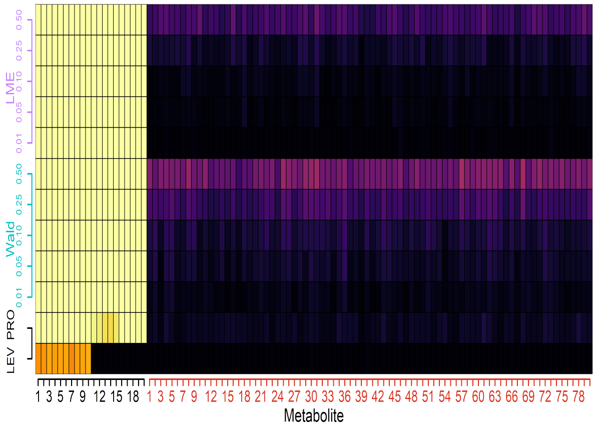


(b) Y generated on levels-scale from cubic spline data, 20 targets and 80 noise

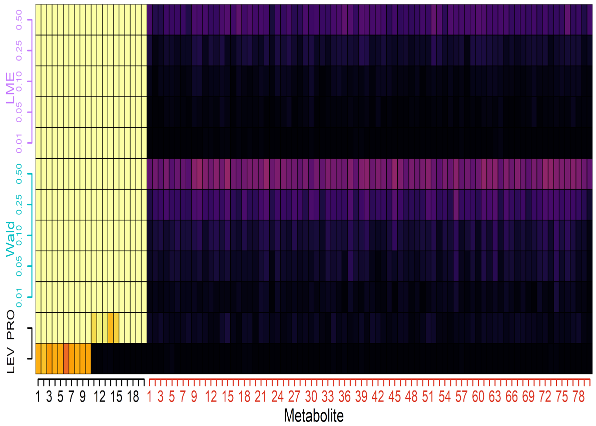


(c) Y generated on levels-scale from cubic spline data with time intercept, 20 targets and 80 noise

Figure S4: Comparison of selection counts (top axis) across simulations for target and noise metabolites (x-axis) using PROLONG, a levels scale version of PROLONG (LEV), univariate Wald tests, and univariate Linear Mixed Effects (LME) models at Various FDR Thresholds (y-axis). The levels-scale PROLONG (LEV) is again significantly worse than PROLONG at identifying targets in all scenarios.

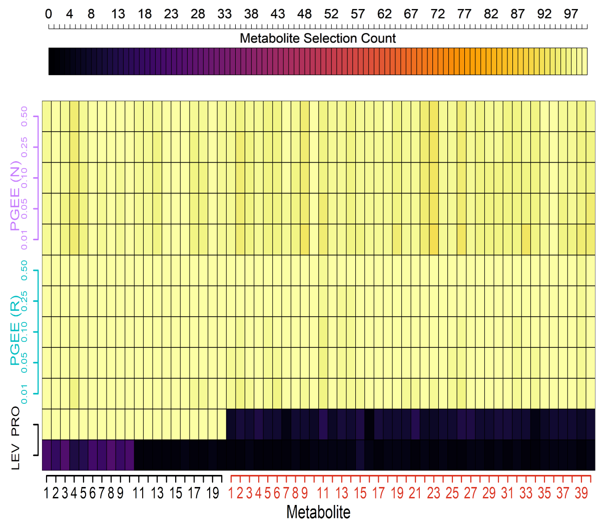


(a) Simulated delta-scale correlated data with no lags, 20 targets and 40 noise

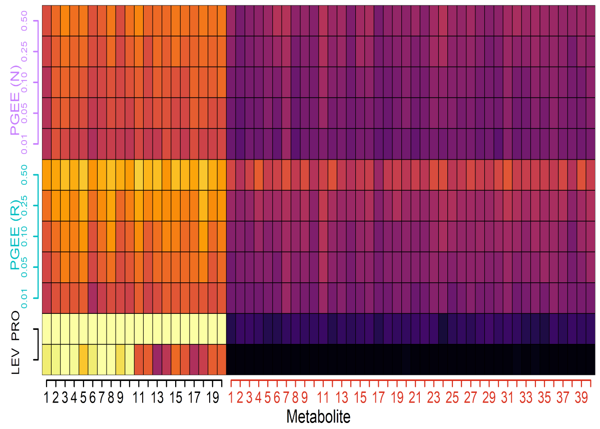


(b) Simulated levels-scale correlated data, 20 targets and 40 noise

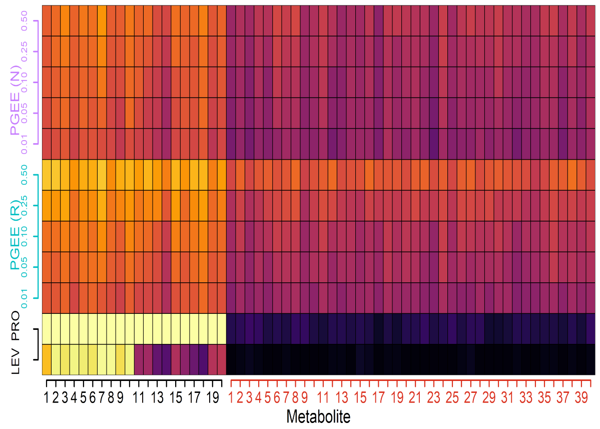


(c) Simulated levels-scale correlated data with time intercept, 20 targets and 40 noise

Figure S5: Comparison of selection counts (top axis) across simulations for target and noise metabolites (x-axis) using PROLONG, a levels-scale version of PROLONG (LEV), and PGEE with both Robust (R) and Naive (N) standard error estimates. The levels-scale PROLONG (LEV) is again worse than delta-scale PROLONG at identifying targets while PGEE, also on the levels-scale, is significantly worse than both at identifying targets failing to separate targets from noise.

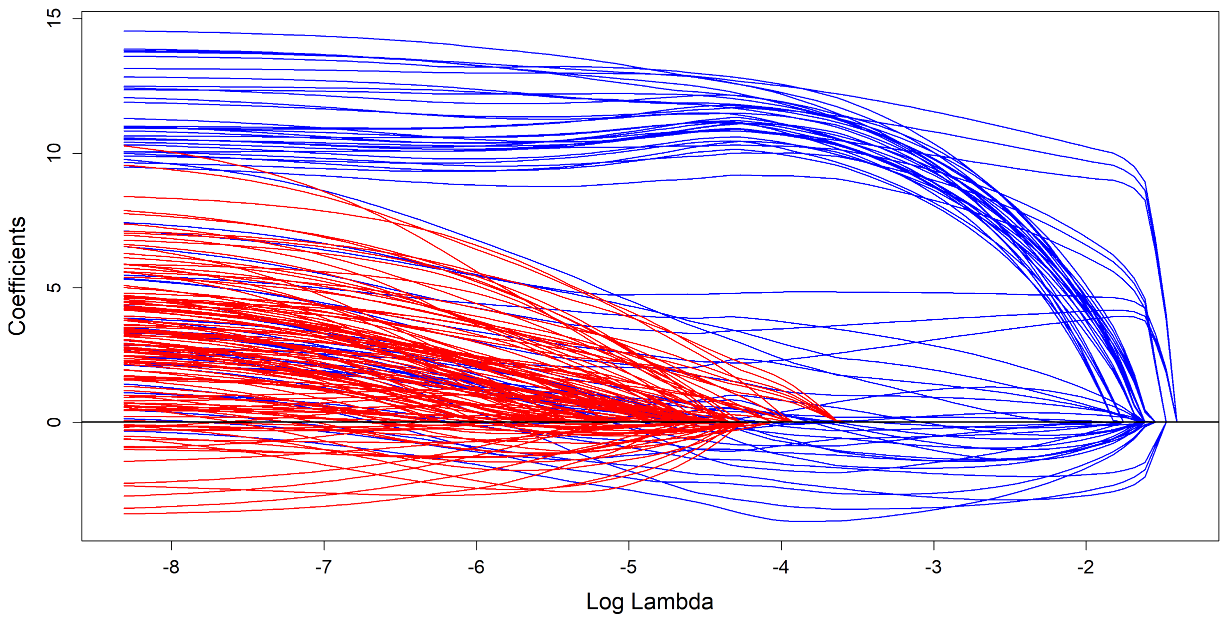


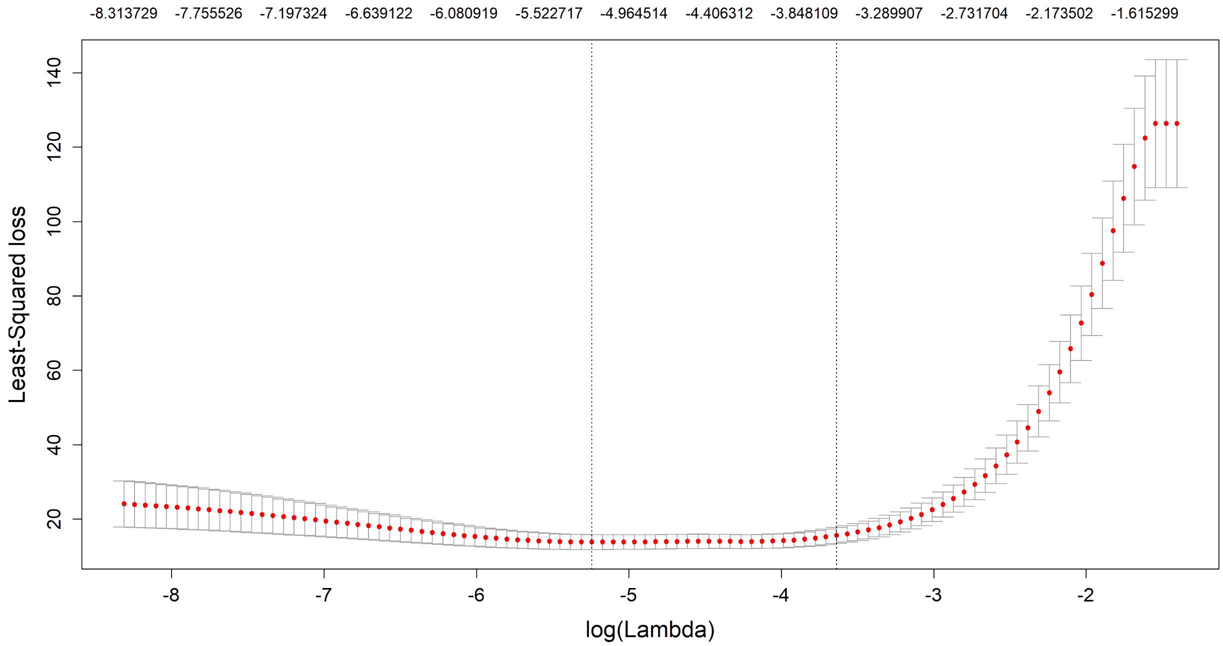


Figure S6: Top image shows coefficient trajectories across group lasso $\lambda_1$ values for both target (blue) and noise (red) simulated metabolites. The bottom image shows LS loss as a function of the group lasso $\lambda_1$. The true metabolites show stable coefficients over $\lambda_1$ and show clear separation in shrinkage compared to the noise.

# References


Wenjiang Fu and Keith Knight. Asymptotics for lasso-type estimators. *The Annals of Statistics*, 28(5), October 2000. ISSN 0090-5364. doi: 10.1214/aos/1015957397.

C. Geyer. On the asymptotics of convex stochastic optimization. *Technical Report. University of Minnesota*, 1996.

Caiyan Li and Hongzhe Li. Network-constrained regularization and variable selection for analysis of genomic data. *Bioinformatics*, 24(9):1175–1182, May 2008. ISSN 1367-4803. doi: 10.1093/bioinformatics/btn081.

Yuval Nardi and Alessandro Rinaldo. On the asymptotic properties of the group lasso estimator for linear models. *Electronic Journal of Statistics*, 2:605 – 633, 2008. doi: 10.1214/08-EJS200.

Robert Tibshirani, Michael Saunders, Saharon Rosset, Ji Zhu, and Keith Knight. Sparsity and smoothness via the fused lasso. *Journal of the Royal Statistical Society Series B: Statistical Methodology*, 67(1):91–108, 2005.

Hui Zou and Trevor Hastie. Regularization and variable selection via the elastic net. *Journal of the Royal Statistical Society: Series B (Statistical Methodology)*, 67(2):301–320, 2005. ISSN 1467-9868. doi: 10.1111/j.1467-9868.2005.00503.x.
